# Description of the first Spanish case of Gerstmann–Sträussler–Scheinker disease with A117V variant: clinical, histopathological and biochemical characterization

**DOI:** 10.1007/s00415-022-11051-9

**Published:** 2022-03-16

**Authors:** Hasier Eraña, Beatriz San Millán, Carlos M. Díaz-Domínguez, Jorge M. Charco, Rosa Rodríguez, Irene Viéitez, Arrate Pereda, Rosa Yañez, Mariví Geijo, Carmen Navarro, Guiomar Perez de Nanclares, Susana Teijeira, Joaquín Castilla

**Affiliations:** 1 Prion Research Lab, Basque Research and Technology Alliance (BRTA), Center for Cooperative Research in Biosciences (CIC BioGUNE), Derio, Spain; 2 Atlas Molecular Pharma S.L., Derio, Spain; 3 Grupo de Enfermedades Raras y Medicina Pediátrica, Instituto de Investigación Sanitaria Galicia Sur (IISGS), Vigo, Spain; 4 Servicio de Neurología, Complejo Hospitalario de Ourense, Ourense, Spain; 5 Molecular (Epi)Genetics Laboratory, Araba University Hospital, Bioaraba Health Research Institute, Vitoria-Gasteiz, Spain; 6 Animal Health Department, NEIKER-Instituto Vasco de Investigación y Desarrollo Agrario, Basque Research and Technology Alliance (BRTA), Derio, Spain; 7 IKERBASQUE, Basque Foundation for Science, Bilbao, Spain; 8 Centro de Investigación Biomédica en Red de Enfermedades Infecciosas (CIBERINFEC), Carlos III National Health Institute, Madrid, Spain

**Keywords:** Prion disease, Transmissible spongiform encephalopathy, Gerstmann–Sträussler–Scheinker disease, Neurodegeneration, Inherited prion disease, Neuropathology

## Abstract

Gerstmann–Sträussler–Scheinker disease (GSS) is a rare neurodegenerative illness that belongs to the group of hereditary or familial Transmissible Spongiform Encephalopathies (TSE). Due to the presence of different pathogenic alterations in the prion protein (PrP) coding gene, it shows an enhanced proneness to misfolding into its pathogenic isoform, leading to prion formation and propagation. This aberrantly folded protein is able to induce its conformation to the native counterparts forming amyloid fibrils and plaques partially resistant to protease degradation and showing neurotoxic properties. PrP with A117V pathogenic variant is the second most common genetic alteration leading to GSS and despite common phenotypic and neuropathological traits can be defined for each specific variant, strikingly heterogeneous manifestations have been reported for inter-familial cases bearing the same pathogenic variant or even within the same family. Given the scarcity of cases and their clinical, neuropathological, and biochemical variability, it is important to characterize thoroughly each reported case to establish potential correlations between clinical, neuropathological and biochemical hallmarks that could help to define disease subtypes. With that purpose in mind, this manuscript aims to provide a detailed report of the first Spanish GSS case associated with A117V variant including clinical, genetic, neuropathological and biochemical data, which could help define in the future potential disease subtypes and thus, explain the high heterogeneity observed in patients suffering from these maladies.

**Supplementary Information:**

The online version contains supplementary material available at 10.1007/s00415-022-11051-9.

## Introduction

Transmissible spongiform encephalopathies (TSE) are invariably fatal neurodegenerative diseases that affect several mammals, including humans. Also known as prion diseases, is a group of phenotypically variable disorders characterized by their unusual etiology, being an aberrantly folded isoform (PrP^Sc^ or prion) of the physiological or cellular prion protein (PrP^C^), the only component of the causative agent [1]. The accumulation of PrP^Sc^ in the Central Nervous System (CNS) leads to a rapid neuronal death. At the microscopic level, neuronal loss, reactive astrogliosis, formation of PrP^Sc^ aggregates, and in some disease subtypes spongiosis, are the main pathological hallmarks of this group of devastating diseases [2]. In humans, TSE encompass Creutzfeldt–Jakob disease (CJD) and its variant form (vCJD), kuru, fatal insomnia (FI), Gerstmann–Sträussler–Scheinker disease (GSS) and variably protease-sensitive prionopathy (VPSPr). Some of them can be sporadic, in which PrP^C^ misfolds into PrP^Sc^ putatively in a spontaneous manner [sporadic CJD (sCJD), sporadic FI (sFI) and VPSPr]. Others can be acquired from exogenous sources such as contaminated food, administration of contaminated human growth hormone or surgical procedures [vCJD, iatrogenic CJD (iCJD) and kuru]. In addition, others are genetic or associated with pathogenic variations in the prion protein-coding gene (*PRNP*) which seem to favor PrP^C^ misfolding [familial or genetic CJD (fCJD/gCJD), familial FI (FFI) and GSS] [3].

The three autosomal dominant genetic human prion diseases are caused by different alterations of the *PRNP* gene. Each of them presents particular clinical and pathological hallmarks, although phenotypic variability is also usual among patients with the same disorder [4]. Among them, GSS encompasses the most diverse clinical spectrum. Usually characterized by prominent cerebellar ataxia between forty and sixty years accompanied by gradually progressive cognitive decline, it can also manifest as an isolated cognitive impairment similar to Alzheimer’s disease [5]. Neuropathological findings are also quite diverse although the most characteristic feature is the presence of multicentric amyloid plaques derived from abnormal PrP products that are mainly distributed in the cerebral cortex, basal ganglia, and cerebellum. Unusual characteristics include the presence of neurofibrillary tangles formed of hyperphosphorylated tau, which have been reported in some GSS cases linked to specific pathogenic variants [5–8]. The exact incidence of the disease is unknown since familial clusters are not systematically reported. Many alterations have been associated with GSS, being P102L the most frequent (of almost 400 of GSS cases reported, around 250 present P102L pathogenic variant) and the first one associated with the disease. However, many other missense variants, few nonsense alterations (coding for an early stop codon) and insertions in the octapeptide repeat region of the protein have also been associated with this disorder [6]. Each variant results in a particular disease phenotype and neuropathological alterations, although variability has been also reported for inter-familial cases bearing the same pathogenic variant or even within the same family [9–11]. Finally, the biochemical features of the PrP^Sc^ found in GSS cases are also unique and distinct from those of other human prion diseases. This consists of C- and N-terminally truncated protease-resistant PrP fragments ranging between 6 and 11 kDa accompanied in some cases by a variable number of bands of higher molecular weight [12].

Given the small number of cases and the high variability, they present at clinical, neuropathological and biochemical levels, reporting each case is important to get the whole picture of GSS and clearly define commonalities and differences between patients. Herein, we report the first GSS case with A117V pathogenic variant from a Spanish citizen and the sixth of all GSS cases found until now in the country [13]. From all GSS cases reported worldwide, A117V always associated with Valine polymorphism at position 129 is the second most common after P102L, with about 40 cases reported worldwide. This genetic alteration was first described in an Alsatian patient [14] and was readily confirmed as a GSS-associated mutation by clinical and genetic studies in other members of the same family [10, 15]. Since then, GSS cases with A117V variant have been reported also in other families in France, Germany, Hungary, the United Kingdom, Ireland, the United States of America and Argentina [6, 11, 16–20]. According to the last report of the Epidemiological Surveillance service from the Spanish Government that encompasses all the reported cases of prion disorders in Spain from 1993 to 2018, there were 5 GSS cases during this period (3 confirmed and 2 probable). The one presented here is the sixth one, and the second reported with A117V mutation, but the first found in a Spanish citizen. The 5 cases of GSS constitute 0.26% of all the cases of human TSE reported in the country during this period. This reflects the extremely low prevalence of this disease even among human TSE [13].

Given the scarcity of cases worldwide, the most complete possible case reports including biochemical characterization of PrP^Sc^, are of great importance to gain insight on the pathobiology of these highly variable disorders. Therefore, we report an utter case study of the first Spanish GSS-A117V patient, including clinical, genetic, neuropathological and biochemical data.

## Materials and methods

Samples and data of the patient included in this study were provided by the Biobank at the Galicia Sur Health Research Institute and they were processed following standard operating procedures with the appropriate approval of the Ethics and Scientific Committees.

### Clinical information

The patient was examined at the Neurology Service of the University Hospital of Ourense. Clinical observations and tests described in “Results” section were performed in the same Hospital complex, including cerebrospinal fluid (CSF) extraction for the determination of 14-3-3 protein levels, neuroimaging and electroencephalogram (EEG).

### Genetic studies

Genomic DNA was extracted from blood for the genetic study of a set of genes associated with Alzheimer’s disease, other tauopathies and Huntington disease. Subsequently, genomic DNA extracted from frozen brain tissue was used to sequence the full-length coding region of the *PRNP* gene, and an allele-specific PCR was performed. See technical details in Supplementary information.

### Neuropathology

The autopsy was carried out five hours after death. The right hemisphere was cut into coronal sections, frozen on dry ice, and stored at − 80 °C until use, while the left hemisphere was fixed in 4% buffered formalin for 15 days and used for microscopic examination.

After fixation, coronal sections of the left hemisphere were treated with formic acid and then post-fixed in formalin and embedded in paraffin. Then, cut at 5 µm and stained with hematoxylin and eosin, Congo Red and Thioflavin S. Immunohistochemistry was carried out using the following primary antibodies against: PrP (3F4, Dako) (with pretreatment with PK), β-amyloid 1–40 and β-amyloid 1–42 (from Dr M. Sarasa, Zaragoza, Spain), hyperphosphorylated tau (AT8; Innogenetics), 3R tau (RD3, clone 8E6/C11, Merck) (1:3000) and 4R tau (RD4, clone 1E1/A6, Merck) (1:100), glial fibrillary acidic protein (GFAP) (EP672Y, Roche), CD68 (KP-1, Roche) and α-synuclein (Chemicon).

### Electron microscopy

Electron microscopy examination was performed on selected fragments from cerebral and cerebellar cortices after glutaraldehyde fixation and routine embedding in Epon. Semi-thin sections were stained with toluidine blue and PAS [21]. Contrasted ultra-thin sections were examined with a Philips CM100 transmission electron microscope. Electron micrographs were taken as part of the medical records.

### Detection and biochemical analysis of PrP^Sc^

10% (w/v) homogenates of frozen samples from the frontal cortex, temporal cortex, occipital cortex, parietal cortex, thalamus, cerebellum and basal ganglia were used for biochemical PrP^Sc^ detection and analysis. Three different assays were performed for all brain homogenate samples: PrP^Sc^ detection and characterization by immunoblotting and epitope mapping, PrP^Sc^ detection by Real-Time Quaking-Induced Conversion assay (RT-QuIC) and PrP^Sc^ detection by Antigen-capture enzyme immunoassay (EIA). See technical details in Supplementary information.

## Results

### Clinical findings

The patient was a 43-year-old male who presented cognitive impairment showing apathy, irritability and language alterations as initial signs in 2006. According to his relatives, he was showing talking difficulties (slowed speech, occasionally unintelligible), apathy and irritability 3 years before admission to the Neurology Service. Already a year before, he started showing difficulties for daily life tasks (slow reactions while driving, carelessness with personal hygiene, lack of interest for his offspring and other social relationships and activities, difficulties to perform his job, especially for multitasking and occasionally childish, obsessive and repetitive behavior). Upon first neurological evaluation, Mini-mental state examination (MMSE) indicated mild cognitive impairment and bradyphrenia, attention and concentration impairment, and episodes of unmotivated laughing were also detected. Other signs of cognitive impairment included altered digit span, reduced verbal fluency, severe phonological alteration, abstraction inability and pathological Trail Making. Further exploration also revealed other neurological disturbances such as reduced facial expressivity and primitive reflexes (glabellar and palmomental). Cerebral perfusion single photon emission computed tomography (brain perfusion SPECT) was performed showing impairment of the perfusion at the fronto-parietal level with a greater impact on the right hemisphere and associated with ventricular dilatation. Nuclear magnetic resonance also showed cerebellar atrophy and diffuse cortical–subcortical atrophy. Within a year, the patient started showing difficulties swallowing and an electroencephalography (EEG) performed during this first year showed a delta wavelength focus at the frontotemporal region with more accused left projection. After a year and a half from initial diagnosis, the neurological status of the patient worsened significantly, showing greater cognitive and motor impairment and requiring assistance for daily activities. He manifested loss of interest on his surroundings and loss of initiative as well as gait disturbances, agitation and restlessness. One of the possible causes of his rapid deterioration was the prescription of neuroleptic medication (haloperidol), which was substituted by quetiapine, which improved the motor activity. A second EEG performed in this period showed acute theta wavelength focus at the frontotemporal region with more accused left projection. Two years after initial diagnosis the patient was hospitalized again due to tonic and clonic movements of the limbs and generalized stiffness, stertorous breathing and subsequent sleepiness. Upon neurological examination, he evidenced a severe language impairment (able to speak few isolated words), generalized muscular atrophy and complete loss of walking ability, as well as sleep disorders with episodes of insomnia. At this point, 14-3-3 protein levels were determined providing negative results. In 2010, he was hospitalized again due to a respiratory infection and vomits, showing a highly deteriorated general condition (emaciation, breathing difficulties and enteral nutrition required). In this final stage, the patient presented mutism, paralysis and dementia with occasional tonic and clonic crisis, and after several respiratory infections, he finally died because of this cause, 6 years after the onset of the disease and diagnosed as probable rapidly progressing FTD or possible CJD.

### Family history

**Fig. 1:**
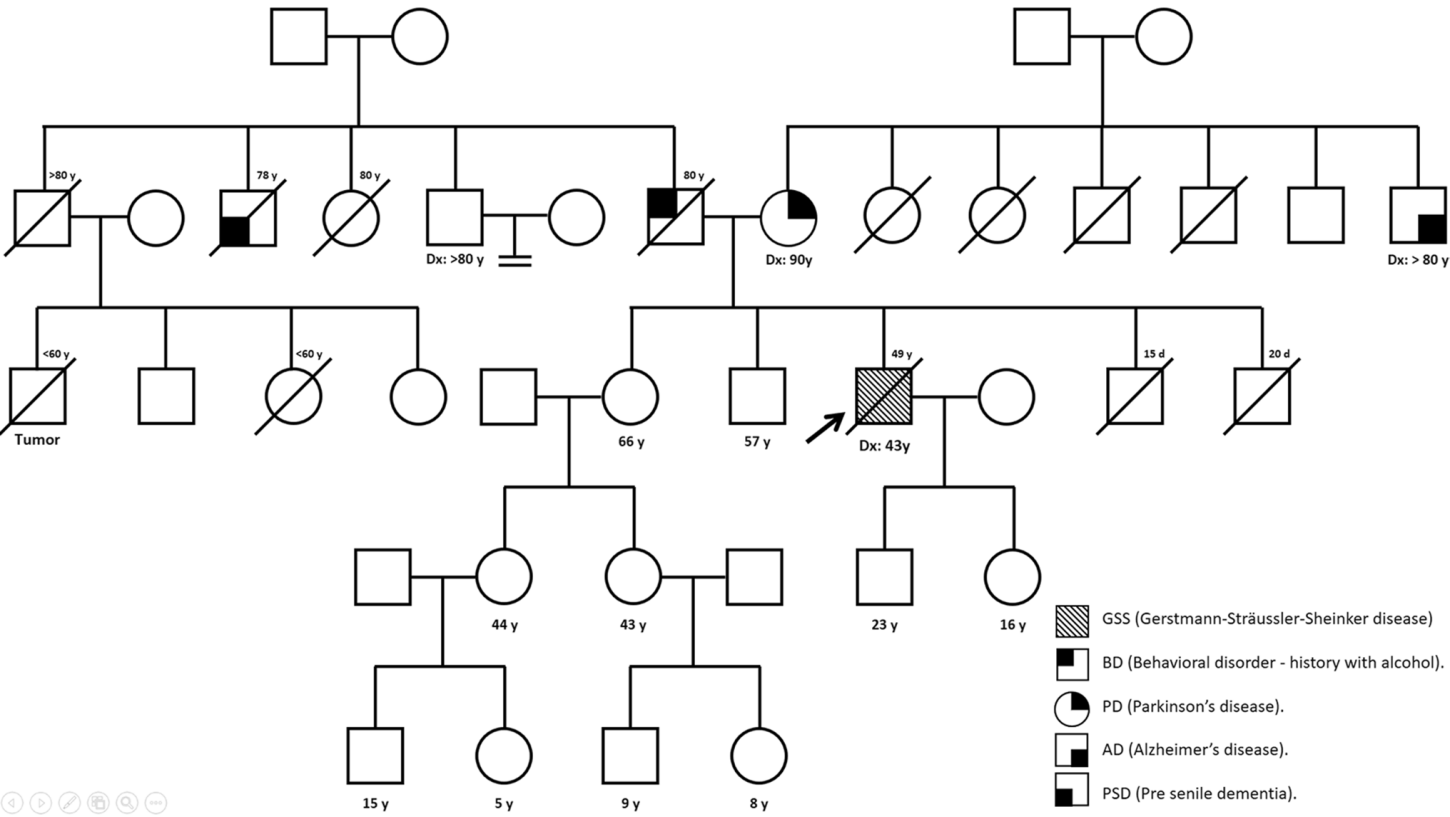
Family history of the patient (indicated with an arrow) noting any neurological disorder, including pre-senile dementia, behavioral disorders, Parkinson’s disease and Alzheimer’s disease. Each disease is represented by different symbols according to the legend and age at disease onset indicated as Dx in those cases for which it was known

Family history included few cases of neurological diseases (Fig. 1). The father of the patient died at the age of 80 and suffered from behavioral disorder likely associated with alcohol consumption, and one of his siblings died diagnosed with pre-senile dementia at the age of 78. The mother was diagnosed at the age of 90 with Parkinson’s disease, and one of her brothers was diagnosed from Alzheimer’s disease when he was older than 80. There is a possibility that the pre-senile dementia diagnosed to the patients’ uncle was a misdiagnosis for a prion disorder; however, no other neurological disease was detected in the paternal side.

### Genetic studies

Initially, genetic analysis of genes associated with Alzheimer’s disease, other tauopathies and Huntington’s disease were performed, and none showed any pathology-associated alterations. Upon autopsy, a genetic study of the *PRNP* gene was performed revealing that the patient was heterozygous for the GSS-associated A117V pathogenic variant. Moreover, it showed an A117A polymorphism and was heterozygous M/V for codon 129. Finally, an allele-specific PCR revealed that the V allele in position 117 was in *cis* with the V129 allele (see Supplementary Fig. 1).

### Neuropathology

**Fig. 2:**
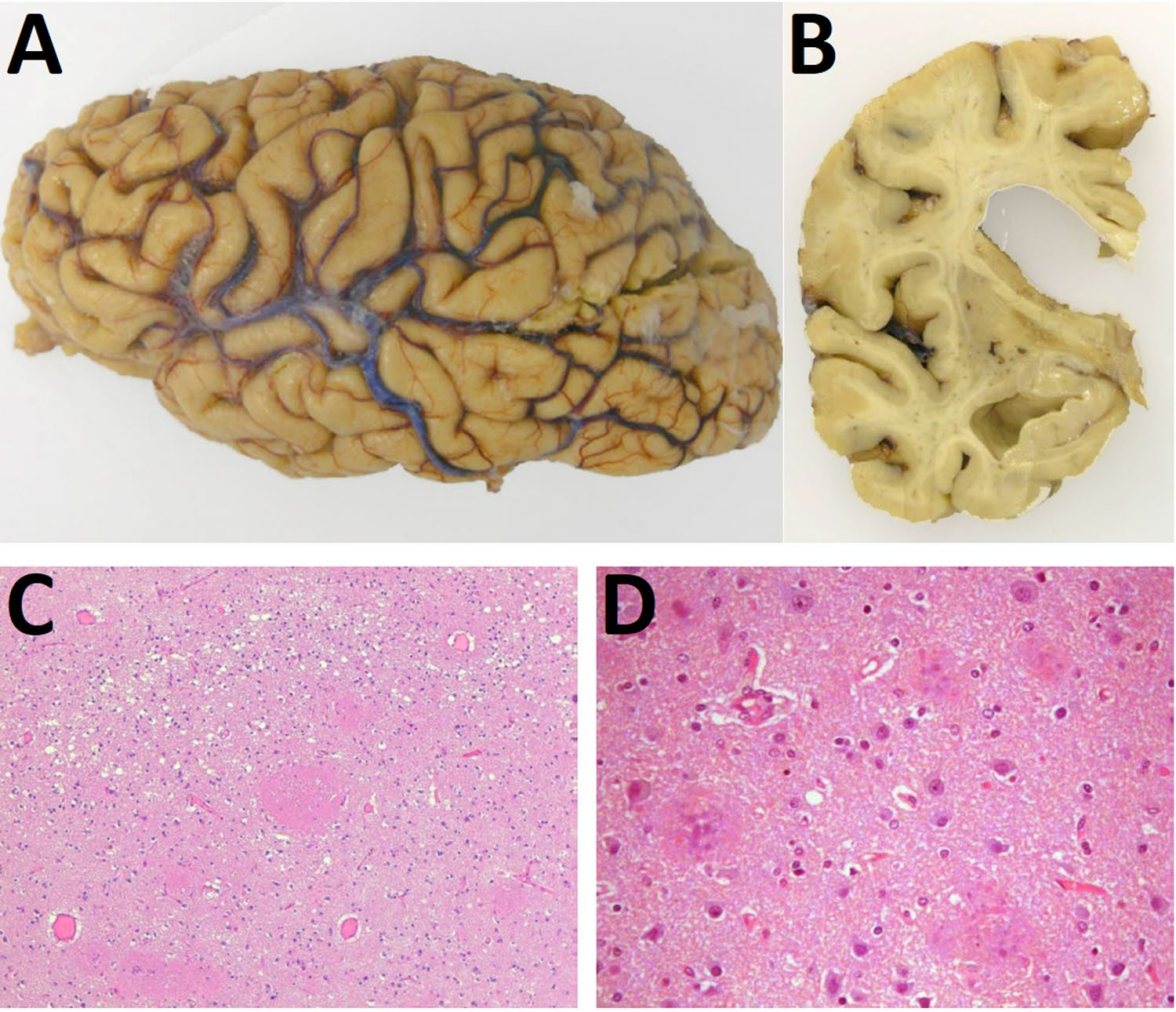
Macroscopic examination of the brain showing **A** cortical brain atrophy partially sparing the occipital lobe and **B** coronal slide demonstrating severe cortical and subcortical atrophy, and subsequent enlargement of the of the frontal and temporal horns of the lateral ventricles. Microscopic anatomopathological analysis of the brain showing **C** spongiosis, gliosis and neuronal loss in the frontal cortex of the brain, being the larger plaques of PrP evident on routine hematoxylin and eosin stains, and **D** details (10X) of the cortical plaques with a dense core

The brain weighed 1050 g, and the macroscopic examination revealed diffuse atrophy of the frontal, temporal and parietal lobes (Fig. 2A). Atrophy was remarkable on coronal slides in the cortical ribbon, the white matter and the basal ganglia, with dilatation of the lateral ventricles (Fig. 2B). Moderate atrophy of the brainstem and cerebellum was also noticeable. On microscopic examination, neuronal loss and astrocytic gliosis were the main findings (Fig. 2C) in the cortical and subcortical gray matter, both in cerebrum and cerebellum. Spongiform cortical changes were mild and no degeneration of the corticospinal tracts was observed. Abundant round unicentric plaques with dense core were detectable upon hematoxylin–eosin staining in the gray matter (Fig. 2D).

**Fig. 3:**
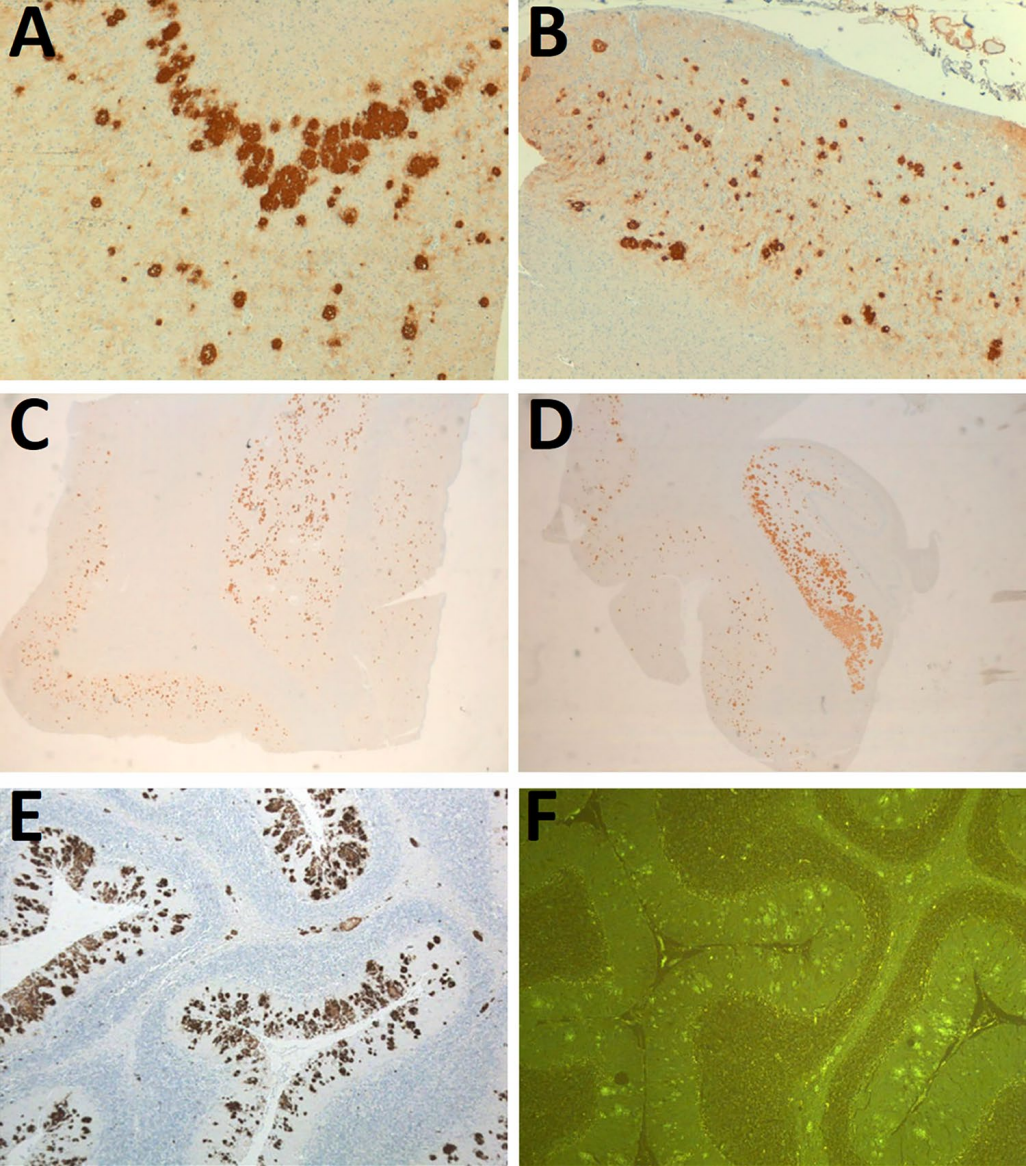
Proteinase K-resistant prion protein (PrP^res^) forming multicentric plaques visualized through immunohistochemistry for PrP (3F4 monoclonal antibody) in the **A**, **B** cerebral cortex, **C** hippocampus, **D** subcortical nuclei (putamen) and in molecular layer and white matter of the cerebellar cortex. Additionally, the plaques were shown to be fluorescent **F** using Thioflavin S staining

PrP immunohistochemistry showed large unicentric and multicentric PrP-amyloid plaques throughout the cerebral and cerebellar cortices, more numerous in deep cortical layers (Fig. 3A, B). The highest density of prion plaques was found in cerebellum and hippocampus, where neighboring plaques converged in an almost continuously stained area, especially in subiculum and pyramidal cell layers CA_1_ and CA_2_ (Fig. 3C, D). Plaques were also abundant in entorhinal and transentorhinal cortices. Regarding the subcortical level, large PrP plaques were present in putamen (Fig. 3E) and thalamus, whereas globus pallidus, caudate and brainstem, showed moderate affectation. The PrP plaques further examined were positive for Congo red staining and showed fluorescence upon Thioflavin S addition (Fig. 3F), demonstrating their amyloidogenic nature.

**Fig. 4:**
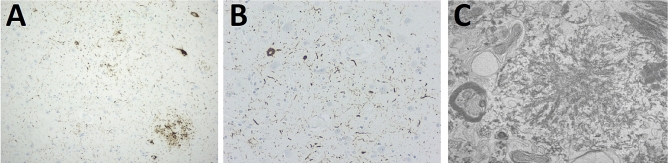
Hyperphosphorylated tau analyzed through immunohistochemistry (AT8 monoclonal antibody) showing tau protein deposits as neutrophilic threads, neuritic plaques and tangles in **A** entorhinal and **B** transentorhinal cortices. **C** Micrograph from transmission electron microscopy showing a PrP^res^ plaque with a complex structure, with a dense fibrillary center from which 8–10 nm fibrils irradiate

Tau protein deposits were found in neutrophilic threads, neuritic plaques and tangles exclusively in the entorhinal and transentorhinal cortices (Fig. 4A–B). The frontal and temporal cortices showed sparse neutrophilic threads, without plaques or tangles. Despite detection of hyperphosphorylated tau, further analysis of these deposits with antibodies specific for 3R and 4R isoforms were negative (data not shown). No co-localization of tau protein or Aβ peptide with the multicentric PrP plaques was observed. No Aβ peptide or α-synuclein was observed in all the locations examined. Immunostaining with CD68 evidenced a marked diffuse microgliosis and a dense infiltrate of foam histiocytes in white matter.

Ultrastructural analysis, performed by Transmission Electron Microscopy (TEM), revealed 8–10 nm fibrils irradiating from the dense center of the plaques (Fig. 4C).

### Biochemical characterization of misfolded protease-resistant PrP

The analysis of proteinase K-resistant PrP in the distinct brain regions by Western blotting and epitope mapping aimed to confirm prion infection, as well as characterizing the PK-resistant core of the prions from this patient.

**Fig. 5:**
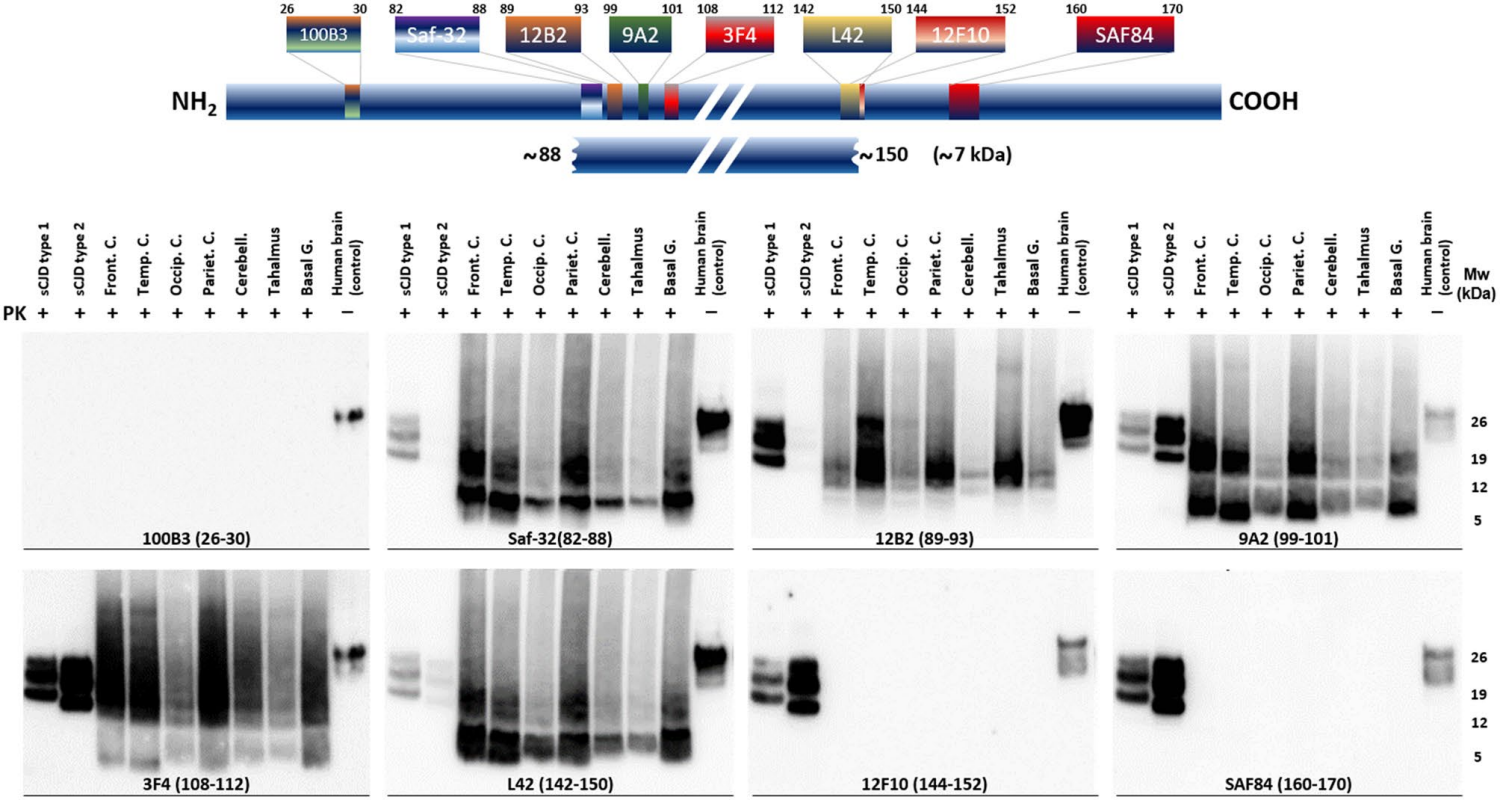
Biochemical characterization of misfolded protease K (PK)-resistant PrP from the brain of the patient using Western blot and epitope mapping to determine the identity of protease-resistant PrP fragments. The blue bar on the top represents the full-length, cellular prion protein from residue 1 at the N-terminus (NH_2_) to the residue 253 at the C-terminus (COOH), with all the different epitopes for the antibodies used represented according to their location within the sequence. The small blue bar below represents the predominant PrP^res^ fragment of approximately 7 kDa, spanning from residues ~ 88–150 according to the epitope mapping analysis shown below. The eight Western blots at the lower part show the PrP^res^ detected on different brain areas of the patient (Front. C.: Frontal cortex; Temp. C.: Temporal cortex; Occip. C.: Occipital cortex; Pariet. C.: Parietal cortex; Cerebell.: cerebellum; Thalamus and B. Ganglia: Basal ganglia) after PK digestion and developed with a different anti-PrP antibody each (100B3, Saf-32, 12B2, 9A2, 3F4, L42, 12F10 and SAF84), indicated in the figure together with the corresponding epitope in agreement with human PrP numbering. In all gels, samples from brain homogenates from sporadic Creutzfeldt–Jakob disease (sCJD) patients with molecular subtypes 1 and 2 and an undigested brain homogenate from a healthy patient (Human brain control) were included as controls. *Mw* molecular weight marker

The most prominent proteolytic fragment is approximately 7 kDa, showing a ladder-like pattern of bands of higher molecular weight (Fig. 5). 100B3 antibody (epitope residues 26–30) with epitope closer to the N-ter could not bind to the PK-digested misfolded PrP, being the Saf-32 (epitope residues 82–88) the most N-ter antibody among those used, able to detect the most prominent ~ 7 kDa fragment and a ladder-like pattern showing bands of approximately 14 and 21 kDa compatible with multimers of the predominant fragment. 12B2 and 9A2 antibodies (epitope residues 89–93 and 99–101, respectively) revealed a similar electrophoretic mobility pattern, although some differences could be observed in the width and intensity of the lower-molecular weight PrP fragment suggesting the presence of ragged ends or slightly different PrP fragments of around 7 kDa. 3F4 (epitope residues 108–112) and L42 antibodies (epitope residues 142–150) also showed a band of approximately 7 kDa, and in the case of the 3F4, bands with higher molecular weight were intensely labeled, higher than the potential dimers and trimers previously observed with Saf-32. On the contrary, L42 shows a double band on the 7 kDa fragment region reminiscent of the wider signals seen with 12B2 and 9A2 antibodies, as well as the same multimers detected with Saf-32. Finally, the most C-ter antibodies used, 12F10 and Saf-84 (with epitope residues 144–152 and 160–170, respectively) did not show any band, indicating that all the PK-resistant PrP fragments observed previously correspond to a region spanning residues ~ 88–148. This fragment, with a theoretical molecular weight of 7.3 kDa (according to Expasy ProtParam), matches the weight of the lower band determined by the molecular weight marker included in each electrophoresis gel.

The distinct brain areas from the patient were also analyzed using two alternative methods for the detection of misfolded PrP, to test if this PrP^Sc^ with atypical electrophoretic pattern could be detected through antigen-capture enzyme immunoassay (EIA) and through Real-Time Quaking-Induced Conversion assay (RT-QuIC). PrP^Sc^ could be detected using both methods, although EIA assay showed lower sensitivity since positive results were obtained only for frontal and temporal cortices (Supplementary Fig. 2), whereas all brain areas were positive by RT-QuIC (Supplementary Fig. 3) (for further details, see Supplementary information). Unfortunately, CSF from the patient was not available at the time of RT-QuIC analysis, so it could only be performed on brain samples.

## Discussion

The rarity of GSS together with the highly variable neuropathological and clinical features that each patient presents makes especially important to report each individual case in the most detailed way possible to further characterize this devastating neurodegenerative disease and reduce the probable misdiagnoses. Moreover, biochemical characterization of the PrP^Sc^ present in each patient, aiming to the establishment of potential disease subtypes could also be useful in that sense, and could explain the phenotypical variability repeatedly reported in GSS cases despite sharing the same alteration [9–11, 22, 23]. From all the changes associated with GSS, around 40 cases bearing A117V variant have been reported, although detailed analysis of PrP^Sc^ has been performed only for few of them.

Among possible clinical phenotypes, there are those more closely related to “pure” dementia showing behavioral and personality disturbances (i.e., mood swings, aggressive behavior, paranoia) which are known as “telencephalic type” and do not manifest the cerebellar ataxia characteristic of many other GSS cases [16]. Other reports also agreed on predominant cognitive signs at onset as characteristic of GSS A117V, although phenotypic variability was evidenced with three sets of clinical signs: i) pure dementia, ii) dementia followed by hemiparesis and pseudobulbar syndrome and iii) dementia with the appearance of more complex signs associated with cerebellar syndrome, extrapyramidal syndrome and motor symptoms [10]. The case reported here is reminiscent of this latter type in which behavioral changes and cognitive decline characterize the onset but with motor symptoms in the last stages of disease. Interestingly, in this case and many others with A117V variant, behavioral changes and cognitive decline frequently present long before other neurological signs, facilitating misdiagnosis with psychiatric conditions and other neurodegenerative disorders such as Alzheimer’s disease [6, 16, 18, 24]. This telencephalic phenotype was wrongly associated with A117V *PRNP* variant at the beginning, in contrast to the ataxic form, which was thought to be linked to P102L alteration. However, in other individuals, the initial manifestation includes variable degrees of cerebellar ataxia, extrapyramidal signs with Parkinsonism features, followed by pyramidal signs, amyotrophy, myoclonus, emotional lability and a pseudobulbar syndrome [6, 11, 19].

Neuropathology and biochemical characteristics of the prion protein deposits are also variable among the individuals affected by GSS with A117V-129V haplotype. Variable amounts of multicentric and diffuse PrP^Sc^ plaques are present in most cases in cerebral cortex, thalamus, basal ganglia and cerebellum, although cases in which no PrP^Sc^ was detected have also been reported [25, 26]. However, the variability appears to be lower than that corresponding to the phenotypic diversity and could not be correlated with the different manifestations such as those showing pure dementia, the ones with parkinsonian features or those presenting with ataxia [11, 18]. In this regard, this case that presented with dementia but develop motor symptoms in later stages of disease, also complied with previous reports showing diffuse cortical– subcortical atrophy, and characteristically, only mild spongiosis. PrP-amyloid deposits were abundant in cortical regions with plaques of variable morphology from diffuse to round multicentric plaques. In addition, the cerebellum was heavily affected, showing high plaque density. This might be one of the notable anatomopathological findings on this case, since cerebellum in GSS associated with A117V variant, is reported to be normally spared [6, 9, 11], although more recent reports have demonstrated it can show variable amounts of PrP deposits in some patients [18, 19]. The presence of neurofibrillary tangles is also remarkable given that, to our knowledge, it has been reported in just one other patient with A117V variant [15], although the lack of complete case reports with thorough histopathological analysis may have precluded such findings in other affected individuals. Nonetheless, based on previous case reports, it seems a rare characteristic, albeit it could be attributed to the age of the patient or the disease duration as was suggested for the previous reported case. In fact, tau deposits in the hippocampus have been described in patients over 40 years old and therefore, the ones present in this patient could respond to a primary age-related tauopathy or initial stages of Alzheimer’s disease [27]. In agreement with this hypothesis, and as found also for some Alzheimer’s disease cases, specific staining with anti-tau 3R or 4R antibodies resulted negative [28], suggesting tau inclusions in this case could be immature, masking the specific epitopes for the two isoforms tested and precluding conclusions on the main isoforms constituting the aggregates. The possibility of coexistence of Alzheimer’s disease and GSS in this patient is unlikely since Aβ immunostaining was negative, but concomitance with other tauopathies could be possible.

Upon digestion with proteinase K (PK), the PrP^Sc^ associated with GSS and A117V variant, if present, shows a ~ 7 kDa internal PrP fragment, usually another one of ~ 14 kDa, and likely less intense, higher molecular weight fragments mainly composed by different multimers of the same PK-resistant protein fragment, in agreement with the results of the biochemical analysis from this case. Previous cases published including thorough biochemical analysis of such fragments, showed they encompass N- and C-terminal ragged ends spanning from residues 86 and 90 to 148, 152, and 153 [12]. Others reported ragged N-terminus, from residues 90–97 ending at residue 146 [29]. The latest study, performed by mass spectrometry-based sequencing, shows three main fragments of ~ 7 kDa, with ragged ends from 78/82/85–88 to 141–152; of ~ 16–18 kDa and ~ 22–23 kDa being trimers and tetramers of the lowest fragment encompassing residues within 82–150 region [30]. In this case, there could be some slight difference on the length of the predominant fragment of around 7 kDa, although it is difficult to confirm such differences due to the ragged N-ter and C-ter ends, also reported in all previous cases. Comparing all of them, it seems that at least two different biochemical signatures for the PrP^Sc^ associated with A117V GSS might exist, one characterized by a larger core encompassing residues 78–153 (around 7–8 kDa) and another one with a smaller PK-resistant fragment of approximately 5 kDa spanning from residues 90–97 to 146. Whether these slightly different biochemical signatures imply the existence of distinct strains or disease subtypes for GSS A117V needs to be further investigated, and the existence of at least these two types of PrP^Sc^ will also require confirmation, since variations in the experimental conditions and in the methods used could be responsible of the observed differences. Finally, it is worth mentioning that GSS A117V causing prions from the present case could be detected by other techniques such as antigen-capture enzyme immunoassay and RT-QuIC, as previously reported for other two cases [31], clearly stating that GSS diagnosis is not limited to histopathological examination and immunoblotting.

Altogether, apart from reporting in detail the main clinical, histopathological and biochemical features of the first Spanish A117V GSS case, we have tried to illustrate the heterogeneity of the cases previously reported with the same pathology-associated alteration to highlight the need of publishing as many cases as possible. Specific clinical and anatomopathological characteristics of each case, such as the presentation with ataxia instead of dementia, or the presence or absence of tau inclusions, together with the biochemical characterization of the PrP^Sc^ in each case may shed some light on the potential existence of different strains responsible for the phenotypic heterogeneity.

## Supplementary Information

Below is the link to the electronic supplementary material.

Supplementary file1 (DOCX 1661 KB)
