# Supplementary material for: Description of the first Spanish case of Gerstmann–Sträussler–Scheinker disease with A117V variant: clinical, histopathological and biochemical characterization
Source: J Neurol. 2022 Mar 16;269(8):4253–63. doi: 10.1007/s00415-022-11051-9 (PMC9293843; doi:10.1007/s00415-022-11051-9)
Supplement: Supplementary file 1 — Supplementary file1 (DOCX 1661 KB) [file 415_2022_11051_MOESM1_ESM.docx]

**Supplemental information**

**Materials and Methods**

*Genetic studies*

Initially, genomic DNA was extracted from blood for the genetic study of a set of genes associated with Alzheimer’s disease (PSEN1 and 2, APP, APO-E), other tauopathies (MAPT) and Huntington disease (HTT). These studies were performed by an external laboratory.

Subsequently, genomic DNA was extracted from frozen brain tissue by the phenol-chloroform method, and the amplification of the full-length coding region of the *PRNP* gene located on the exon 2 (primer sequences available upon request) was performed. The mutational screening was performed by direct sequencing, and the sequences obtained were compared with the reference sequence of the *PRNP* gene (NG_009087.1). This analysis was conducted at the Genomics Unit from the Galicia Sur Health Research Institute.

For the allele-specific PCR, the DNA obtained previously was amplified by allelic-specific oligonucleotide (ASO) polymerase chain reaction method (PCR), to determine if the alteration identified was in *cis* or *trans* phase to 129V. Concretely, the common forward oligonucleotide for both PCR reactions was designed to hybridize upstream to A117V alteration (FM13_A1: 5’- *TGTAAAACGACGGCCAGT*GGCAACAGTGTTTCTACT -3’, represented in italics the FM13 complementary sequence) and reverse ASO were designed to hybridize on the variable position M129V (R_129M: 5’-ATGGCACTTCCCAGCAT-3’; R_129V:5’-ATGGCACTTCCCAGCAC-3’). After performing the PCR reactions in two independent tubes to amplify each allele, both product were subjected to direct Sanger sequencing, using FM13 primer in standard methods and an ABI 3500 Genetic Analyzer (Applied Biosystems, Foster City, CA, USA).

*PrP^Sc^ detection and characterization by immunoblotting and epitope mapping*

Frozen samples of the frontal cortex, temporal cortex, occipital cortex, parietal cortex, thalamus, cerebellum and basal ganglia were homogenized at 10 % (w/v) in 100 mM Tris-HCl at pH 7.4 using 2 mm zirconia beads and a TissueLyser LT (Qiagen). PrP^Sc^ purification for immunodetection was done based on the method developed by Wenborn and colleagues [1]. Briefly, 200 µl of each homogenate were digested with Pronase E (from *Streptomyces griseus*, Sigma-Aldrich) at 100 µg/ml for 30 min at 37 °C and 800 rpm in a Thermomixer (Eppendorf). After digestion, EDTA (Sigma-Aldrich) was added to each sample at a final concentration of 10 mM, as well as sodium lauroyl sarcosinate (sarkosyl) (Sigma-Aldrich) for a final concentration of 2 % (w/v) and Benzonase® (Sigma-Aldrich) at 50 U/ml and were incubated for 10 min. Then, sodium phosphotungstate octadecahydrate (NaPTA) (Sigma-Aldrich) was added to the samples for a final concentration of 0.3 % (w/v), which were incubated for 30 min at 37 °C and 800 rpm. After adding OptiPrep^TM^ (60 % iodixanol density gradient medium) (Sigma-Aldrich) up to 35 % (w/v) and NaPTA to keep the 0.3 % concentration, samples were centrifuged for 90 min at 16 100 *g* (Sorvall ST 16 R, ThermoFisher Scientific). The supernatant was carefully collected and transferred to new tubes after filtration with microcentrifuge filtration units, Ultrafree-HV (0.45 μm pore, Millipore). Supernatants were thoroughly mixed 1:1 with a solution containing 2 % sarkosyl and 0.3 % NaPTA and centrifuged for other 90 min at 16 100 *g*, discarding supernatant and keeping the pellet afterwards. Pellets were resuspended in washing buffer [composed of 17.5 % (w/v) iodixanol and 0.1 % (w/v) sarkosyl] and digested for 1 h at 37 °C and 800 rpm with Proteinase K (PK, Roche) at 10 µg/ml. Digestion was stopped with phenylmethanesulfonyl fluoride (PMSF) (Sigma-Aldrich) at a final concentration of 1 mM and samples were diluted 1:10 with washing buffer. After addition of NaPTA for a final concentration of 0.3 % (w/v) samples were centrifuged for 30 min at 16 100 *g*. Supernatants were discarded and the pellets were washed with 200 µl of washing buffer, to which NaPTA was added for a final concentration of 0.3 % (w/v). After an additional centrifugation for 30 min at 16 100 *g*, supernatants were again discarded and pellets resuspended in 15 µl of NuPAGE 4X loading buffer (Invitrogen), previously diluted 1:3 in PBS (Fisher Bioreagents). Samples were then submitted to SDS-PAGE electrophoresis and Western blotting as described elsewhere [2] and transferred to PVDF membranes (Bio-Rad). Membranes were developed with the following antibodies for the characterization of the PK-resistant fragments: 100B3 (epitope covering residues 26 to 30) (dilution 1:10 000) (Central Veterinary Institute part of Wageningen UR; Lelystad), SAF32 (epitope covering residues 82 to 88) (dilution 1:200) (Cayman Chemical), 12B2 (epitope covering residues 89 to 93) (dilution 1:2500) (Central Veterinary Institute part of Wageningen UR; Lelystad), 9A2 (epitope covering residues 99 to 101) (dilution 1:4000) (Central Veterinary Institute part of Wageningen UR; Lelystad), 3F4 (epitope covering residues 108 to 112) (dilution 1:10 000) (Sigma-Aldrich), L42 (epitope covering residues 142 to 150) (dilution 1:400) (R-Biopharm AG), 12F10 (epitope covering residues 144 to 152) (dilution 1:1000) (Cayman Chemical) and SAF84 (epitope covering residues 160 to 170) (dilution 1:400) (Cayman Chemical). This set of antibodies was chosen to ensure detailed coverage of the N-terminal region and the central part of the PrP, based on previous reports of the main protease-resistant PrP fragment found in GSS patients bearing A117V mutation [3-5], and therefore define potential differences with respect to the main proteolytic fragment determined for this case.

*Real-Time Quaking-Induced Conversion assay (RT-QuIC)*

RT-QuIC assays for disease-associated prion aggregate detection was performed as previously described [6] with minor modifications. Bank vole PrP was chosen as substrate due to the lack of detection of biochemically atypical prions such as those causing GSS by other substrates traditionally used for RT-QuIC [6]. Briefly, bank vole PrP coding sequence (23-231, I109 polymorphism) cloned into a pOPINE expression vector was transformed in *E. coli* BL21 DE3 (Thermo Scientific) which were grown in Luria-Bertoni broth (Pronadisa) medium in the presence of 50 µg/ml ampicillin sodium salt (Sigma-Aldrich). Recombinant protein expression was induced by addition of IPTG (Gold biotechnology) and purification from inclusion bodies was performed under denaturing conditions using Ni-Sepharose columns (His-Trap FF, GE Healthcare) for IMAC with an ÄKTA Start liquid chromatographer (GE Healthcare). The protein was re-folded on the column using a decreasing gradient of guanidine HCl and eluted using an imidazole gradient as described previously [7]. The eluted protein was dialyzed into 10 mM sodium phosphate buffer (pH 5.8), filtered through 0.22-μm syringe filters (Corning), concentration adjusted to 1 mg/ml using centrifugal filter units (Amicon ultra-15 10 KDa, Millipore) and stored at -80°C. RT-QuIC reaction mix were prepared in 10 mM phosphate buffer (pH 7.4), with 300 mM NaCl, 0.1 mg/ml recombinant PrP, 10 μM thioflavin T (ThT) and 1 mM ethylenediaminetetraacetic acid tetrasodium salt (EDTA). 98 μl of the reaction mix were loaded into each well of a black 96-well plate with a clear bottom (Nunc) and seeded with 2 μl of indicated BH dilutions, homogenized at 10 % in PBS and further diluted serially in PBS to reach the indicated dilutions. The plate was then sealed with a plastic film (Nalgene Nunc International) and incubated at 42° C in a BMG FLUOstar Omega plate reader (BMG Labtech) with cycles of 1 min shaking (700 rpm double orbital) and 1 min rest throughout the indicated incubation time. Every 45 min, ThT fluorescence was measured (450 +/-10 nm excitation and 480 +/-10 nm emission; bottom read). Data sets were normalized to a percentage of the maximal fluorescence response of the plate reader after subtraction of the baseline, and plotted versus reaction time.

*Antigen-capture enzyme immunoassay*

All frozen brain region samples were tested using the Bovine Spongiform Encephalopathy-Scrapie Antigen Test Kit, EIA (IDEXX) according to the manufacturer’s protocol for nervous tissue following the ultra-short procedure. Antibodies provided with the kit were substituted by 3F4 (Sigma-Aldrich) primary antibody (dilution 1:5000) and recombinant anti-mouse IgG secondary antibody conjugated with HRP (m-IgGκ BP-HRP, Santa Cruz biotechnology) (1:1500). Previous homogenization of the distinct brain regions at 10 % (w/v) in PBS buffer was performed using a Tissuelyser LT (Qiagen) and zirconia beads of 1 mm diameter (BioSpec Products, Inc.). Samples were considered positive if the optical density values of both replicates were greater than the manufacturer’s cut-off criterion.

**Results**

*Other methods for the detection of misfolded protease-resistant PrP*

The distinct brain areas from the patient were also analyzed using two alternative methods for the detection of misfolded PrP, in order to test if this PrP^Sc^ with atypical electrophoretic pattern could be detected through antigen-capture enzyme immunoassay (EIA) and through RT-QuIC. In the first case, Bovine Spongiform Encephalopathy-Scrapie Antigen Test Kit, EIA from IDEXX with the appropriate modifications for the detection of human prions was used. With sCJD MM1 and VV2 subtypes as positive controls and brains from two different healthy individuals as negative controls, PrP^Sc^ could only be detected in frontal and temporal cortices in agreement with the threshold indicated by the manufacturer’s protocol (Supplementary Fig. 2). Nonetheless, the OD values were much higher for the classical prions included as positive controls than for any of the brain regions with prions associated with A117V GSS. Similarly, although negative according to the threshold established by the manufactures, the rest of the brain regions from the patient with GSS associated to the A117V variant show higher OD values than the negative controls. This demonstrated that albeit with lower sensitivity than for other prions as those causing sCJD, GSS prions can be also detected through EIA.

The analysis of the distinct brain regions of the patient by RT-QuIC using a substrate composed by recombinant bank vole prion protein permitted the detection of misfolded PrP in all brain areas tested. As observed in the EIA assay, positive controls consisting on brain homogenates of sCJD patients with MM1 and VV2 subtypes required much shorter assay times (about 10 h) to induce misfolding of the recombinant PrP from the substrate, while for GSS prions positive signals were detected between 55 and 80 h (Supplementary Fig. 3). However, since the brain homogenate from a healthy individual and another one with Alzheimer’s disease used as negative controls did not induce PrP misfolding in more than 90 h of reaction, it can be concluded that RT-QuIC is suitable for the detection of A117V GSS causing prions.

**Supplementary figures**

**
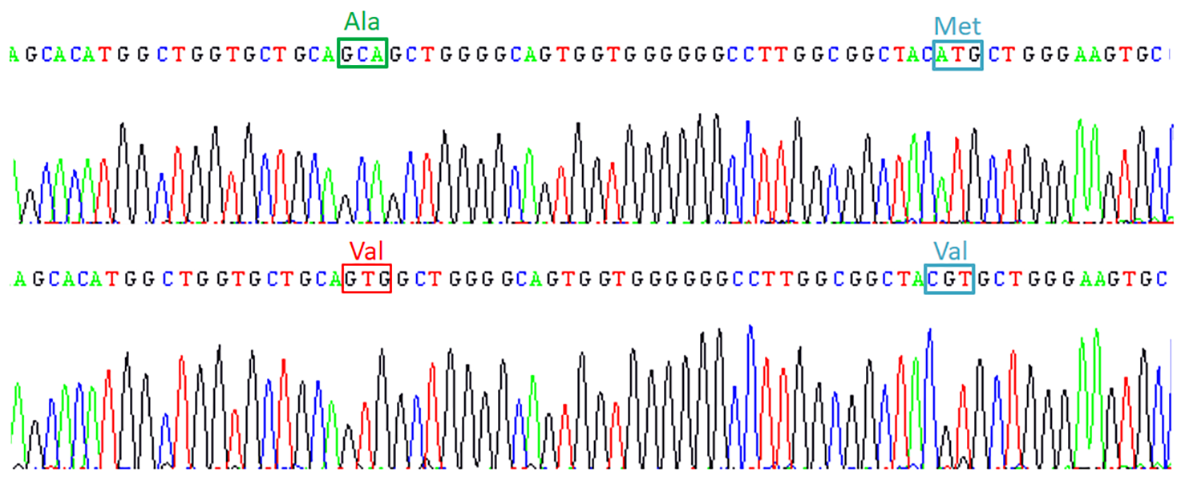
**

**Supplementary Fig. 1** Electropherogram revealing the sequence obtained after the allelic –specific PCR. The upper panel shows the sequence for the A allele (corresponding to the M129 variant), whereas the bottom panel corresponds to C allele (V129)

**
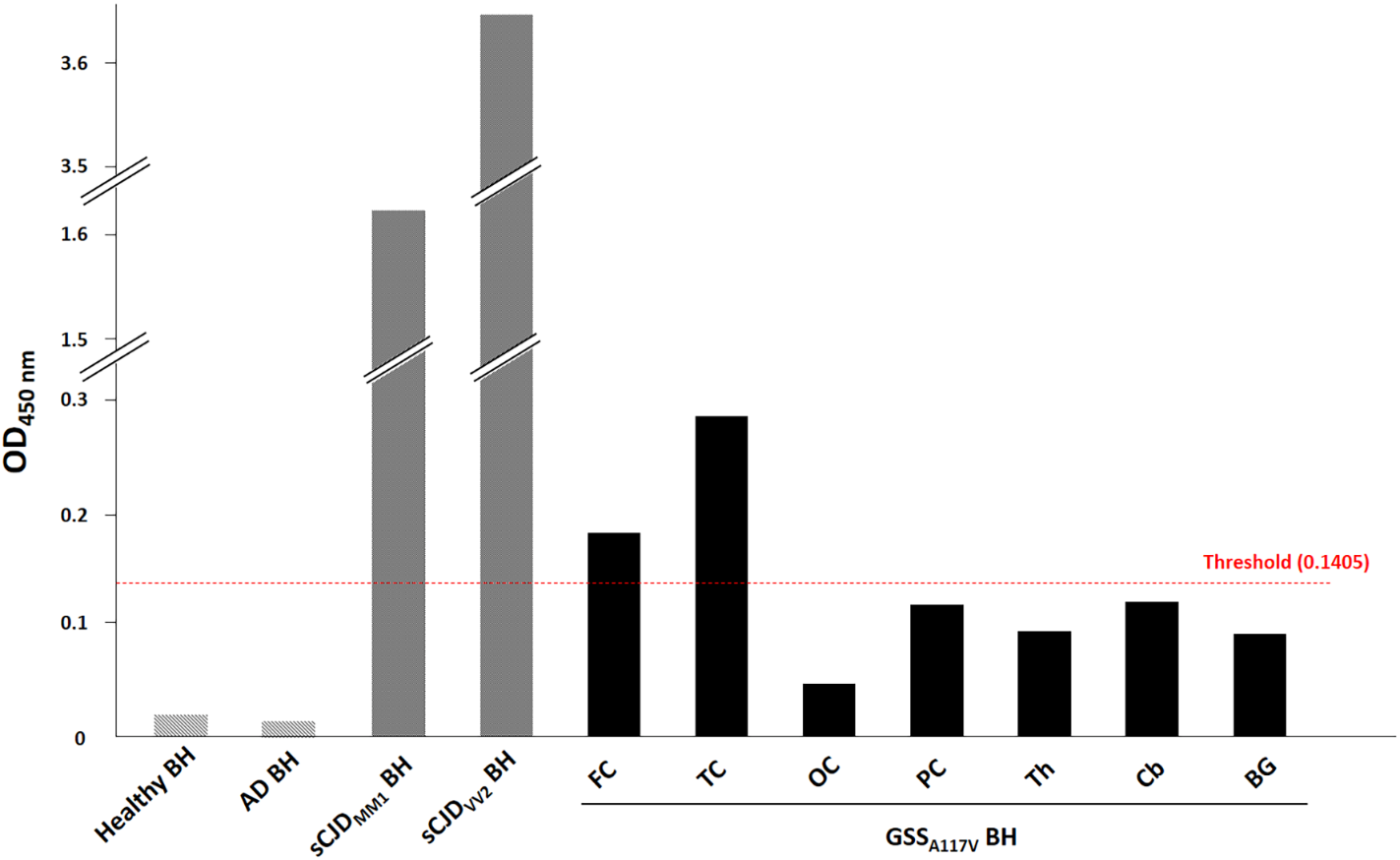
**

**Supplementary Fig. 2** Graphical representation of the results from antigen-capture enzyme immunoassay (EIA) for the detection of misfolded prion protein. Using the Bovine Spongiform Encephalopathy-Scrapie Antigen Test Kit from IDEXX with appropriate modifications for human PrP detection, seven different brain areas from the patient were analyzed (FC: Frontal cortex; TC: Temporal cortex; OC: Occipital cortex; PC: Parietal cortex; Th: Thalamus; Cb: Cerebellum; BG: Basal ganglia) and absorbance at 450 nm measured. The threshold above which a sample can be considered positive for prions, according to the kit’s manufacturer’s instructions, is indicated with a dashed red line. As controls, a brain homogenate (BH) from a healthy donor was included, as well as, a brain homogenate from a patient with Alzheimer’s disease (AD BH) and from patients with two different sporadic CJD subtypes (sCJD_MM1_ BH and sCJD_VV2_ BH). Although which much lower signal than sCJD prions, GSS prions could be detected using this technique in at least two brain areas, frontal and temporal cortices, likely related to a higher PrP^res^ amount. Despite being below the threshold, the rest of the brain areas from the same patient show higher signal than the negative controls used in the study

**
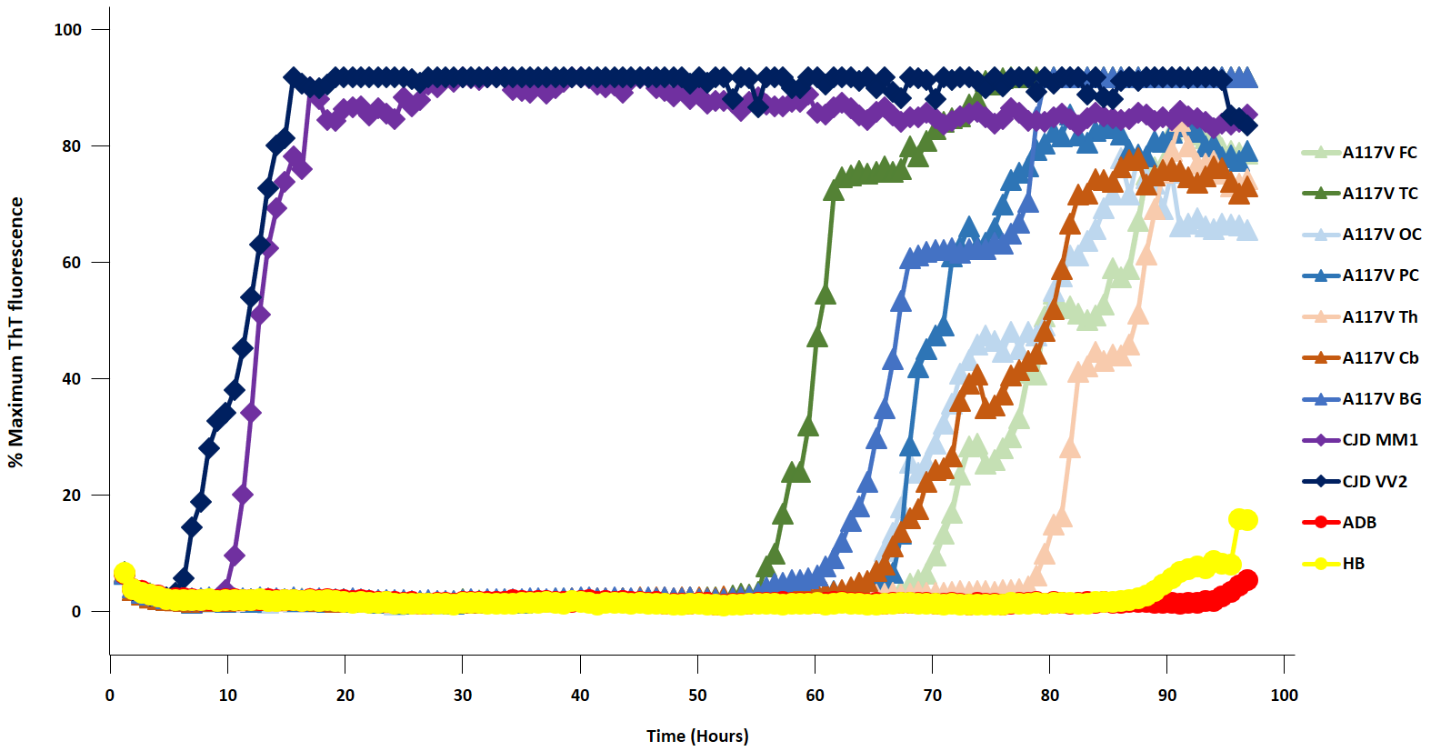
**

**Supplementary Fig. 3** RT-QuIC detection of misfolded prion protein in different areas of the central nervous system of the patient with GSS using full-length recombinant bank vole I109I PrP as substrate. RT-QuIC reactions were seeded with 10^-5^ dilutions of brain homogenate from different areas: Frontal Cortex (FC), Temporal Cortex (TC), Occipital Cortex (OC), Parietal Cortex (PC), Thalamus (Th), Cerebellum (Cb) and Basal Ganglia (BG). As positive controls, RT-QuIC reactions were seeded with 10^-5^ dilutions of brain homogenates from confirmed sCJD MM1 and sCJD VV2 patients. As negative controls, RT-QuIC reactions were seeded with 10^-5^ dilutions of a brain homogenates from an Alzheimer’s disease patient (ADB) and a healthy donor (Healthy Brain HB). Despite showing a longer lag phase than brain homogenates from patients with sporadic Creutzfeldt-Jakob disease, GSS causing prions could be also detected in all brain areas analyzed

**References**

1. Wenborn A, Terry C, Gros N, Joiner S, D'Castro L, Panico S, Sells J, Cronier S, Linehan JM, Brandner S, Saibil HR, Collinge J, Wadsworth JD (2015) A novel and rapid method for obtaining high titre intact prion strains from mammalian brain. Sci Rep 5:10062. <https://doi.org/10.1038/srep10062>

2. Erana H, Perez-Castro MA, Garcia-Martinez S, Charco JM, Lopez-Moreno R, Diaz-Dominguez CM, Barrio T, Gonzalez-Miranda E, Castilla J (2020) A Novel, Reliable and Highly Versatile Method to Evaluate Different Prion Decontamination Procedures. Front Bioeng Biotechnol 8:589182. <https://doi.org/10.3389/fbioe.2020.589182>

3. Tagliavini F, Lievens PM, Tranchant C, Warter JM, Mohr M, Giaccone G, Perini F, Rossi G, Salmona M, Piccardo P, Ghetti B, Beavis RC, Bugiani O, Frangione B, Prelli F (2001) A 7-kDa prion protein (PrP) fragment, an integral component of the PrP region required for infectivity, is the major amyloid protein in Gerstmann-Straussler-Scheinker disease A117V. J Biol Chem 276:6009-6015. <https://doi.org/10.1074/jbc.M007062200>

4. Pirisinu L, Nonno R, Esposito E, Benestad SL, Gambetti P, Agrimi U, Zou WQ (2013) Small ruminant nor98 prions share biochemical features with human gerstmann-straussler-scheinker disease and variably protease-sensitive prionopathy. PLoS One 8:e66405. <https://doi.org/10.1371/journal.pone.0066405>

5. Cracco L, Xiao X, Nemani SK, Lavrich J, Cali I, Ghetti B, Notari S, Surewicz WK, Gambetti P (2019) Gerstmann-Straussler-Scheinker disease revisited: accumulation of covalently-linked multimers of internal prion protein fragments. Acta Neuropathol Commun 7:85. <https://doi.org/10.1186/s40478-019-0734-2>

6. Orru CD, Groveman BR, Raymond LD, Hughson AG, Nonno R, Zou W, Ghetti B, Gambetti P, Caughey B (2015) Bank Vole Prion Protein As an Apparently Universal Substrate for RT-QuIC-Based Detection and Discrimination of Prion Strains. PLoS Pathog 11:e1004983. <https://doi.org/10.1371/journal.ppat.1004983>

7. Groveman BR, Kraus A, Raymond LD, Dolan MA, Anson KJ, Dorward DW, Caughey B (2015) Charge neutralization of the central lysine cluster in prion protein (PrP) promotes PrP(Sc)-like folding of recombinant PrP amyloids. J Biol Chem 290:1119-1128. <https://doi.org/10.1074/jbc.M114.619627>
